# Viral Latency in Blood and Saliva of Simian Foamy Virus-Infected Humans

**DOI:** 10.1371/journal.pone.0077072

**Published:** 2013-10-08

**Authors:** Rejane Rua, Edouard Betsem, Antoine Gessain

**Affiliations:** 1 Department of Virology, Unit of Epidemiology and Physiopathology of Oncogenic Viruses, Institut Pasteur, Paris, France; 2 Centre National de la Recherche Scientifique (CNRS), Institut Pasteur, Paris, France; 3 Paris Diderot University, Cellule Pasteur, Paris, France; 4 Department of Microbiology, Parasitology, Hematology, Faculty of Medicine and Biomedical Sciences, University of Yaounde I, Yaounde, Cameroun; Metabiota, United States of America

## Abstract

Simian foamy viruses (SFV) are widespread retroviruses among non-human primates (NHP). SFV actively replicate in the oral cavity and can be transmitted to humans through NHP bites, giving rise to a persistent infection. We aimed at studying the natural history of SFV infection in human. We have analyzed viral load and gene expression in 14 hunters from Cameroon previously shown to be infected with a gorilla SFV strain. Viral DNA could be detected by quantitative polymerase chain reaction (q-PCR) targeting the pol-in region, in most samples of peripheral blood mononuclear cells (PBMCs) (7.1 ± 6.0 SFV DNA copies/105 PBMCs) and saliva (2.4 ± 4.3 SFV DNA copies/105 cells) derived from the hunters. However, quantitative real-time reverse-transcription polymerase chain reaction (RT)-qPCR revealed the absence of SFV viral gene expression in both PBMCs and saliva, suggesting that SFV was latent in the human samples. Our study demonstrates that a latent infection can occur in humans and persist for years, both in PBMCs and saliva. Such a scenario may contribute to the putative lack of secondary human-to-human transmissions of SFV.

## Introduction

Emerging human infections caused by viruses originating in wildlife have increased significantly recently [1] mainly due to more frequent contacts between wild animals and humans for demographical and socio-economical reasons. Viral emergence is a multi-step process involving virus transmission to humans and subsequent human-to-human spread. This latter step depends on the natural history of the virus (including viral load, replication and tropism) within the human host.

SFV are exogenous complex retroviruses of the *Spumaretrovirinae* subfamily. They are ubiquitous in various species including non-human primates, felines, bovines and equines [2]. Furthermore, they induce a life-long, persistent infection without apparent pathogenicity [3].

Previous studies have highlighted important aspects of SFV replication and transmission in NHP. SFV DNA was detected in PBMCs from NHP [4,5], and no viral RNA could be detected, suggesting that PBMCs constitute a site of SFV latency. SFV DNA was also detected in buccal swabs of NHP [6]. Importantly, SFV active replication occurs in a superficial epithelial cell niche of the oral mucosa in naturally SFV-infected NHP, as shown for several captive macaques (*Macaca mulatta*) [5,7]. This is in agreement with the observed transmission of SFV in NHP through bites [8]. It also explains the high prevalence of SFV infection among humans bitten by a monkey or an ape [9].

The viral load of SFV-infected humans remains poorly studied and ranges from 1 to 1755 copies/10^5^ blood cells [10–12].

The presence of SFV in the oral mucosa of SFV-infected humans is still a matter of debate. Schweizer et al. could not isolate SFV from the saliva of a healthy animal caretaker who acquired the virus 20 years before from an African green monkey (AGM) bite [13]. By contrast, Boneva et al. studied in detail 7 individuals living in the USA, occupationally exposed to NHP (mainly working as veterinarians or animal caretakers), and infected by SFV. SFV DNA could be detected by nested-PCR in the saliva of 3/7 SFV-infected individuals and could be isolated from the saliva of 1/6 individual [14].

However, the quantification of both SFV DNA load and SFV RNA load in the saliva of SFV-infected humans is still lacking.

In an area of frequent cross-species transmission, in order to get a better understanding of the natural history of SFV in humans, we aimed at characterizing *in vivo* infections of humans by SFV. Our goals were to:

1. Quantify total SFV DNA load in samples of both PBMCs and saliva derived from persons infected by a gorilla strain of SFV (SFVggo).
2. Assess levels of viral gene expression (SFV RNA) in human PBMCs and saliva samples.

We show that low levels of SFV DNA can be found in samples of PBMCs and saliva derived from SFV-infected hunters. However, no SFV RNA was detected in these samples. This absence of detectable viral replication suggests that SFV infection in humans is latent in both PBMCs and saliva.

## Materials and Methods

### FV status of enrolled individuals

We enrolled in this study 14 participants (Ako394, Bad348, Bad447, Bad456, Bad468, Bak33, Bak56, Bak74, Bak82, Bak132, Bak133, Bak177, Lobak2, Lobak89) who had been shown to be infected with a gorilla SFV strain in our previous work [11] (Table 1). Briefly, the 14 participants were determined infected by SFV based on both serological results (Western-Blot positivity) and molecular studies on PBMCs (FV detection by nested- polymerase chain reaction PCR on the *pol-in* fragment (integrase coding sequence of the polymerase gene) and/or the LTR fragment). All of them have been bitten by a gorilla, which is very probably the cause of SFV infection, as suggested previously [11]. Of note, the strain of SFV infecting each individual always matched with the NHP species they reported being bitten by, declared in the interview.

**Table 1:** Epidemiological features of the 14 SFV-infected humans.

**Individual**	**Ethnicity**	**Age at contact**	**Age at sampling**	**Wound location**	**Severity**
Ako394	Bantu	53	53	Thigh	1
Bad348	Bantu	19	27	Leg	3
Bad447	Bantu	40	56	Hand	2
Bad456	Bantu	24	30	Leg	2
Bad468	Bantu	25	35	Several	3
Bak33	Pygmy	25	45	Multiple	3
Bak56	Pygmy	40	65	Hand	2
Bak74	Pygmy	26	47	Foot	2
Bak82	Pygmy	46	50	Leg	2
Bak132	Pygmy	30	61	Head	3
Bak133	Pygmy	30	51	Several	3
Bak177	Pygmy	26	36	Leg	3
Lobak2	Pygmy	37	57	Thigh	3
Lobak89	Pygmy	20	50	Arm	2

All individuals lived in South Cameroon rainforest villages/settlements, had been bitten by a gorilla during hunting activities and are infected by a gorilla SFV. Age at contact was assumed, based on interview.

Wound severity is based on interview and physical injury, from small scar with little bleeding to profound scar with fracture or loss of finger or leg.

1 = low, 2 = medium, 3 = high.

### Ethics statement

All participants received detailed information about the study and gave written consent. The study received administrative and ethical clearance in Cameroon from the research division of the Ministry of Public Health and from the National Comity of Ethics, and in France, from the «Comité de protection des Personnes» and the «Commission Nationale de l’Informatique et des Libertés».

### Specimen collection and preparation

Blood and saliva samples of each participant were collected on the same day. Fresh blood samples were collected in EDTA tubes and shipped at room temperature from the Pasteur Institute of Yaoundé to the Pasteur Institute of Paris for processing. Within 20 hours following sampling, PBMCs were isolated on a Ficoll-Hypaque gradient (30min, 1800 g). About 0.5 to 2 million PBMCs were frozen for subsequent DNA and RNA extraction. For saliva samples, the 14 participants were asked to rinse their mouth with 2 ml of phosphate buffer saline (PBS). This mouthwash sample was centrifuged (2min,1000g) and the supernatant was discarded. Buccal swabs were also collected by gently touching the oral mucosa with a cotton swab and were diluted in PBS. They were added to the pellet of the mouthwash sample. Centrifugation was performed in the same tube as the mouthwash sample (2min, 1000g). Cell pellets and debris were kept at -80°C in Yaoundé and sent frozen in dry ice to the Pasteur Institute of Paris. Then, they were stored at -80°C for subsequent DNA and RNA extraction.

### DNA and RNA extraction

For the molecular studies, genomic DNA was extracted from PBMCs and saliva samples by using the QIAamp DNA Blood Mini Kit (QIAGEN, Courtaboeuf, France). RNA was extracted from PBMCs and saliva samples using the RNeasy Plus Mini Kit (QIAGEN, Courtaboeuf, France).

### SFV standard curve

To standardize quantitative PCR (qPCR), a 465 bp region that included the PCR target sequence from one primary isolate (BAK74, gorilla strain of SFV) was cloned into a PCR cloning plasmid, TOPO TA cloning kit (Invitrogen). We calculated the standard curve of SFV plasmid as follows:

Number of SFV copies/µl= 6.02 10^23^ x Plasmid concentration (g/µl) / Molecular Weight of SFV plasmid (g/mol)

Molecular Weight (MW) of SFV plasmid= MW of TopoTA cloning vector (2.4 10^6^ g/mol) + MW of SFV *pol-in* insert (0.3 10^6^ g/mol)

We sequenced the SFV plasmid to make sure that only one SFV insert was present in the TopoTA cloning plasmid.

Primers (GF5qpcr-TAGACCTGAAGGAACCAAAATAATTCC, and GR5qpcr-TCCTTCCTCATATTAGGCCACC) were designed to detect a 144 bp nucleic acid region of the gorilla SFV polymerase gene. In each assay, SFV DNA copies from one to 10^4^ were in the range of linear detection, and coefficient correlation of SFV standard curves was >0.95. The standard curve (from 1 to 10^4^ copies) was performed in duplicates, and both duplicates indicated the same number of SFV DNA copies, apart from 1 copy, which was detected in 1/2 or 2/2 replicates.

To confirm the reliability of the standard curve based on SFV plasmid, we constructed a standard curve based on purified SFV *pol-in* PCR products after nested-PCR on BAK74 PBMC DNA. We obtained the same result (1 to 10^4^ SFV DNA copies were detected, and coefficient correlation was >0.95).

### Quantification of SFV DNA in the PBMCs and saliva samples

#### qPCR conditions

Quantitative PCR assays for DNA were performed as previously described [11]. Quantitative PCR assays for DNA (qPCR) were performed using the Eppendorf realplex master gradient detection system. We used SYBR Green Quantitect (Qiagen) in a 20 µl volume reaction containing 10 µl of SYBR Green buffer, 150 nM of each primer and up to 500 ng DNA sample quantified by ND 8000 (*Nanodrop* Technologies).

The optimized qPCR conditions used were as follows: 95°C for 15min, 40 cycles of: 95°C for 15s, 60°C for 30s and 72°C for 30s. Samples were tested at least in duplicates in two independent assays. We checked in every individual assay the specificity of the primers by using a melting curve.

#### Normalization of cellular DNA

The albumin DNA quantification method was the following: a 141bp-cellular albumin qPCR was done performed to normalize with cellular DNA content (albF-AAACTCATGGGAGCTGCTGGTT, albR- GCTGTCATCTCTTGTGGGCTGT) [4], using the same PCR conditions as for SFV quantification.

All assays included an independent albumin standard curve generated by adding 5-fold dilutions of human genomic DNA (fibroblastic MS5 cell-line) to the PCR reagents, ranging from 0.08ng DNA to 50ng DNA. All qPCR standard curves had correlation coefficients of >0.95.

The albumin DNA content of the samples was determined as follows: the amount of DNA was firstly quantified by Nanodrop technologies and samples of 500ng cellular DNA were used. To normalize samples input, a 250x dilution of the samples (2ng) was used. The amount of albumin DNA was determined using the albumin DNA standard curve, reported as cell equivalents (500ng DNA=75 000 cells), and multiplied by 250 to calculate the “effective” amount of cellular DNA in the samples. The minimum amount of “effective” DNA was 350ng DNA (5 x 10^4^ cells equivalent).

#### qPCR sensitivity

FV sensitivity was thus given as 1 copy for 5 x 10^4^ cells equivalent. Of note, for some samples (as AKO394 PBMCs, BAD447 saliva, BAK74 saliva, LOBAK89 PBMCs), the input DNA, after normalization, was >350ng, giving a sensitivity >1 copy for 5 x 10^4^ cells equivalent.

### Quantification of SFV RNA in the PBMCs and saliva samples

#### RT-qPCR conditions

Quantitative real-time reverse-transcription polymerase chain reaction (RT-qPCR) was performed using the same standard as qPCR. RNA samples quantified by ND 8000 (*Nanodrop* Technologies) were mixed with random hexamer primers (5µM final), dNTP (1mM final) and completed with DEPC-treated water until a final volume of 10 µl. The RNA mixture was subjected to denaturation: 75°C for 5 min, following by 4°C for 5 min (Mastercycler, epGradient ; Eppendorf). Ten µl of enzyme mix including the SuperScriptIII® Reverse Transciptase (Invitrogen) were added to the RNA mixture and subjected to 3 steps: 25°C for 10 min, 45°C for 90 min and 9°C for 5 min. cDNA obtained was added to 10 µl of SYBR Green buffer and 150 nM of each GF5qpcr and GR5qpcr primers in a final volume of 20 µl. The optimized RT-qPCR conditions used were as follows: 95°C for 15min, 40 cycles of: 95°C for 15s, 60.8°C for 30s and 72°C for 30s. Samples were tested at least in duplicates in two independent assays. We checked in every individual assay the specificity of the primers by using a melting curve. Our positive control was RNA extracted from the human microglial cell line CHME productively infected with SFV strain from BAK74.

#### Normalization of cellular RNA

The GAPDH cDNA quantification method was the following: a 107bp-cellular GAPDH qRT-PCR was done to normalize with cellular RNA content (GAPDH-F 5′- ATGGGGAAGGTGAAGGTCG-3′ and GAPDH-R 5′- GGGTCATTGATGGCAACAATATC-3′) [15], using the same PCR conditions as for SFV quantification.

All assays included an independent GAPDH cDNA standard curve generated by adding 5-fold dilutions of human genomic cDNA (CHME microglial cell line) to the PCR reagents, ranging from 10pg to 100ng starting RNA. All RT-qPCR standard curves had correlation coefficients of >0.95.

To normalize samples input, a 4x dilution of the samples was used. The amount of GAPDH cDNA was calculated using the GAPDH cDNA standard curve, reported as cells equivalent (50ng RNA= 10^4^ cells), and multiplied by 4 to calculate the effective amount of cellular RNA in the samples. The minimal RNA content was 2.5ng RNA (500 cells equivalent), except for the lowest concentrated samples (100pg RNA, 20 cells equivalent).

#### RT-qPCR sensitivity

The sensitivity of the assay was thus 1 copy/500 cells equivalent, except for the lowest concentrated samples (sensitivity of 1 copy/20 cells equivalent).

### *Pol-in* sequences

A nested-PCR was carried out for the specific detection of SFV DNA using generic primers [16]. *Pol-in* PCR products (465bp-length) were cloned and sequenced as previously described [11]. Sequences after trimming of the PCR primer sequences are 425bp-length. Genetic variation was measured using the consensus sequence of all SFV clones derived from PBMCs and saliva.

### Phylogenetic analyses

For every selected clone, both forward and reverse amplified nucleotide sequences were aligned using ‘‘ClustalW alignment » software included in the DAMBE version 4.5.68 [17]. According to Akaike Information Criterion (AIC), different evolutionary models were tested using PAUP software version 4.0b10 (Sinauer associates, Inc. Publishers, Sunderland, Massachussets). Phylogeny was performed with the neighbor joining method and the best tree was selected after a bootstrap analysis of 1000 replicates. GenBank accession numbers of the gorillas SFV strains used in the multiple alignments are: SFVggo(BANGA) (AY686191), SFVggo(BINTA) (AY686193), SFVggo(CAM7) (AY583782), SFVggo(GORGABCOL) (AY603409), SFVggo(GORGABOMO) (AY603410), SFVggo(KOOB) (AY686194), SFVggo(KUCHI) (AY686192), SFVggo(LP5) (EU527593).

Sequences of the SFV *pol-in* clones from PBMCs and saliva were deposited in GenBank under the accession numbers: KC602129-KC602164 for LOBAK2, KC602201-KC602238 for BAD456, KF515319-KF515357 for BAD447, KF515358-KF515395 for BAK33, KF515396- KF515431 for BAK74, KF515432-KF515469 for BAK132, KF515470-KF515508 for BAK133, KC602165 to KC602200 for BAK177.

## Results

As SFV infection is latent in macaques PBMCs but productive in their oral cavity [5,7], we asked whether this was also the case for SFV-infected humans. To determine the levels of SFV DNA and RNA in the oral cavity of the infected individuals, we used oral swabs, a noninvasive, accessible source of saliva and cellular material.

### SFV DNA is present in most PBMCs and saliva samples derived from infected hunters

#### Quantification of SFV DNA load

We studied 14 SFV-infected hunters, all of them infected by a gorilla SFV strain (Table 1). They have been determined SFV-positive in a previous epidemiological study by both serological and molecular findings [11].

**Figure 1:**
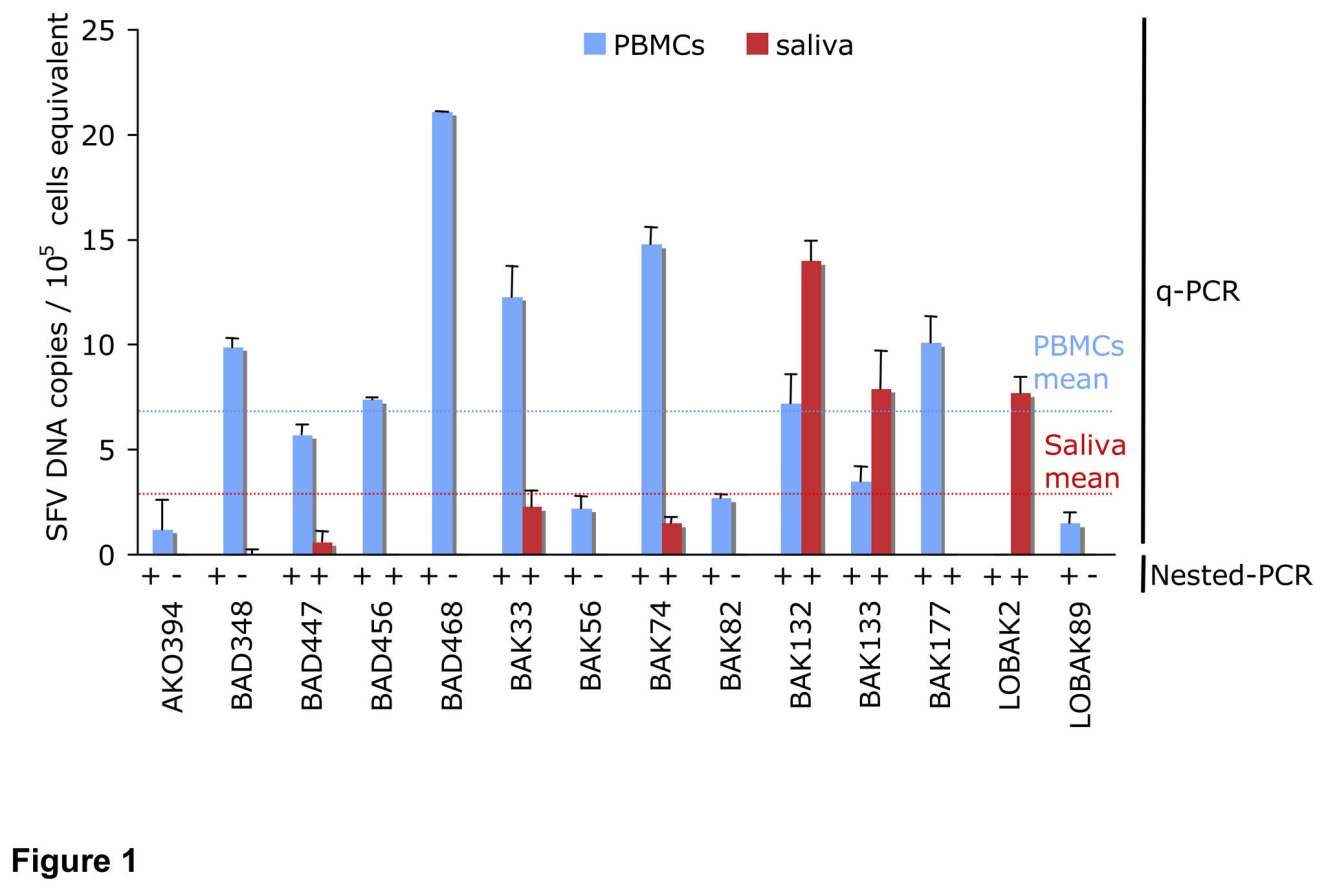
Detection and quantification of Simian Foamy Virus (SFV) DNA in samples of PBMCs and saliva derived from 14 humans infected by a gorilla strain of SFV. Detection of SFV DNA: Presence (+) or absence (-) of SFV DNA in PBMCs and saliva samples of each individual are indicated, based on nested-PCR results. Quantification of SFV DNA: Blue and red bars represent mean SFV DNA levels from at least two independent assays run in duplicate in PBMCs and saliva samples, for each individual respectively. Viral loads were normalized to cell equivalents by q-PCR for albumin. Error bars represent the standard deviations from an individual participant. Mean of SFV DNA copies in PBMCs (blue) and saliva (red) of the 14 individuals are shown by the dotted lines.

We first confirmed the SFV status of the 14 individuals. We performed a nested-PCR on the *pol-in* region of SFV using DNA isolated from PBMC samples (Table 2). As expected, all the individuals were found to be SFV-infected (Figure 1 “Nested-PCR” line under “PBMCs” bars). To explore the presence of SFV DNA in saliva samples, we performed the same nested-PCR on oral swabs. We detected SFV DNA in 8/14 saliva samples (Figure 1 “Nested-PCR” line under “Saliva” bars).

**Table 2:** Availability of cellular DNA and RNA of the PBMCs and saliva samples from 14 SFV-infected individuals.

**Individual**	**Available samples**			
	**Cellular DNA**		**Cellular RNA**	
	**PBMC**	**saliva**	**PBMC**	**saliva**
AKO394	+	+ *	+	+ *
BAD348	+	+	+ *	+
BAD447	+	+	+	+
BAD456	+	+	+	-
BAD468	+	+	NA	+ *
BAK33	+	+	NA	+ *
BAK56	+	+	+	-
BAK74	+	+	NA	+ *
BAK82	+	+	NA	+
BAK132	+	+	+ *	+
BAK133	+	+	+	-
BAK177	+	+	+ *	+
LOBAK2	+	+	+	+ *
LOBAK89	+	+ *	+	+ *

+ presence of DNA or RNA

absence of RNA

NA : not available for RNA extraction

* samples with lower DNA or RNA content (5 000 cells equivalent instead of 50 000 and 20 cells equivalent instead of 500 per well respectively)

We then quantified SFV DNA load by performing q-PCR on the same samples. SFV DNA load could be quantified in 13/14 PBMC samples and in 6/14 saliva samples (Figure 1). Total SFV DNA load in PBMCs and in saliva were very low in most of the samples (7.1 ± 6.0 and 2.4 ± 4.3 SFV DNA copies/10^5^ cells respectively). No correlation was found between SFV DNA loads in the PBMCs and saliva samples Of note, only individuals with long time of infection (>16 years, which is the median) had more than 1 SFV copy/10^5^ cells in the saliva (data not shown).

The sensitivity of the technique used to detect SFV DNA is higher for our nested-PCR assay (1 copy/150 000 cells) [10] than for our q-PCR assay (1 copy/50 000 cells): 3/28 DNA samples (saliva from BAD456 and BAK177 and PBMCs from LOBAK2) were positive by nested-PCR but negative by q-PCR, probably due to a low viral load (Figure 1). Overall, in 8/14 cases, SFV nested-PCR was positive in DNA samples of both PBMCs and saliva derived from SFV infected-individuals (Figure 1 and Table 3).

**Table 3:** Analysis of the SFV quasispecies present in PBMCs and saliva of the 14 SFV-infected individuals.

**Individual**	**Nested-PCR SFV pol**		**Number of SFV clones**	
	**PBMC**	**Saliva**	**PBMC**	**Saliva**
AKO394	+ (1)	- (1)		
BAD348	+ (1)	- (1)		
BAD447	+ (1)	+ (2)	19	20
**BAD456**	**+ (1)**	**+ (1)**	**20**	**18**
BAD468	+ (1)	- (3)		
BAK33	+ (1)	+ (1)	28	10
BAK56	+ (2)	- (2)		
BAK74	+ (1)	+ (1)	19	17
BAK82	+ (1)	- (1)		
BAK132	+ (1)	+ (1)	18	20
BAK133	+ (3)	+ (1)	19	19
BAK177	+ (1)	+ (2)	17	19
**LOBAK2**	**+ (1)**	**+ (1)**	**20**	**16**
LOBAK89	+ (2)	- (2)		

DNA samples were first analyzed by nested-PCR (+: positivity, - negativity; the number of trials is indicated within brackets). When both PBMCs and saliva nested-PCR were positive for *pol-in* PCR products, 10-28 clones were sequenced and analyzed. SFV sequences derived from BAD456 and LOBAK2 displaying G/A polymorphism in the context of a dinucleotide GG are indicated in bold characters.

#### Characterization of SFV clones

**Figure 2:**
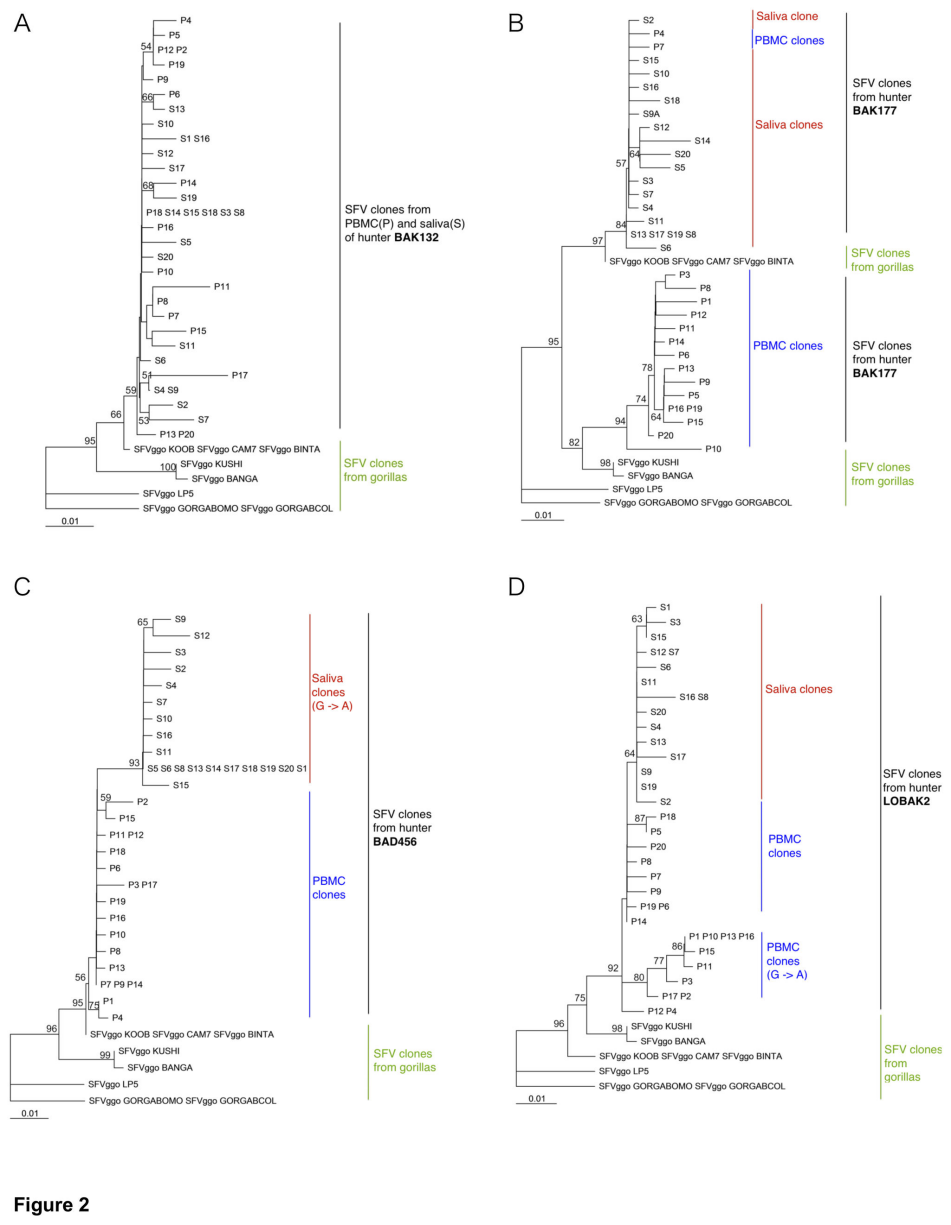
Phylogenetic tree generated with sequences of 425 bp fragments of the SFV *pol-in* from blood and saliva of SFV-infected hunters BAK132, BAK177, BAD456 and LOBAK2 and different gorillas Sequences from hunters BAK132 (A), BAK177 (B), BAD456 (C) and LOBAK2 (D) are indicated in red (saliva clones, "S") and blue (PBMC clones, "P"). Sequences are compared to 8 sequences available in Genbank from different central African NHP gorillas (green). Alignment was performed with the Dambe version 4.5.68 and Clustal W. Phylogenetic analysis was performed with PAUP, version 4.0b10 (Sinauer Associates, Sunderland, MA, USA) based on the Neighbor joining method applying the best model calculated by AIC: HKY (BAK132, BAK177), GTR (BAD456) or TrN (LOBAK2). Bootstrap analysis of 1000 replicates is shown as percentage at nodes. Only values greater than 50% are shown. The scale of the tree is 0.01 nucleotides replacement per site. Of note, phylogenetic tree for BAK132 is representative of trees obtained for the other following hunters (BAK33, BAK74, BAK132, BAD447). Sequences accession numbers for BAK132 SFV clones are KF515432-KF515469. Phylogenetic tree for BAK177 indicates the presence of two distinct groups of SFV clones, suggesting a co-infection with two SFV strains. Sequences accession numbers for BAK177 SFV clones are KC602165 to KC602200. For BAD456 and LOBAK2, some clones displayed G to A mutations (G->A) present in a GG dinucleotide context and absent in the 8 NHP gorillas strains. Sequences accession numbers of SFV clones are KC602201 to KC602238 (BAD456) and KC602129 to KC602164 (LOBAK2). Due to very low viral loads, a single cluster of SFV clones within one compartment (PBMCs or saliva) might derive from PCR amplification of a single SFV copy.

We generated SFV clones of the *pol-in* fragment (mean number of 18 clones) when both saliva and PBMCs samples contained SFV DNA (8/14 individuals). At the individual level, the sequences of SFV clones derived from PBMCs versus saliva often differed by only 0.5% genetic variation (in 5/8 individuals, Figure 2A).

In 1/8 case (BAK177), a higher genetic variation (3.3%) in the range of the inter-strain variability was found among SFV clones which segregated into two clusters (Figure 2B). Such data could indicate a co-infection by two SFV strains.

For individuals BAD456 and LOBAK2, consensus sequences of PBMCs and saliva SFV clones were not identical and displayed a **G/A** polymorphism in a **G**G dinucleotide-context (representing 5/5 and 4/8 nucleotide differences respectively), with the A polymorphism being absent in the 8 SFV strains found in gorillas and available in GenBank (Figures S1 and S2 provide alignments of SFV sequences, and Figure 2C and 2D provide corresponding phylogenetic trees).

### SFV RNA is not detectable in PBMCs and saliva samples from SFV-infected hunters

To determine if PBMCs or cells of the saliva were permissive for SFV infection, we used qRT-PCR targeting viral *pol-in* RNA, as an indication of viral replication. Since primers in the *pol* gene were used, our measurements include both SFV genomic RNA and SFV *pol* transcripts. We checked that this assay could detect SFV RNA from a SFV permissive cell line (the human microglial cell line CHME) infected with the SFV strain from individual BAK74, after appearance of the typical *in vitro* cytopathic effect (data not shown). We next examined PBMCs and saliva samples of 10/14 and 14/14 SFV-infected individuals respectively (4/14 PBMC samples were not available for RNA extraction). Although cellular RNA was detected in all the PBMC samples tested and in 10/14 saliva samples by GAPDH RT-QPCR, no SFV viral RNA was detected in any of these samples (Table 2). As the cell pellet, and not the cell supernatant, was studied, we cannot exclude the presence of SFV viral particles in the saliva, lost upon centrifugation. Though, if particles were to be made, SFV-infected cells undergoing productive infection would have been probably detected by the presence of SFV RNA.

## Discussion

In this study, we report the detection and quantification of SFV DNA in saliva and PBMCs derived from 14 persons infected by SFV of zoonotic origin. However, we found no SFV RNA in these samples and hence, no evidence of viral replication. Our findings concerning SFV RNA in the saliva, which are the very first reported in humans, are in contrast to the findings in SFV-infected NHP, especially concerning the lack of detectable viral RNA in saliva.

These observations raise the following questions concerning the natural history of SFV infection in humans.

### What are the characteristics of SFV DNA in PBMCs and saliva of SFV-infected individuals?

#### SFV DNA load in PBMCs of SFV-infected individuals

In NHP, few studies are available on viral load in blood samples. In 11 healthy, non-immunosuppressed macaques (*Macaca mulatta*), SFV DNA is present at a low copy number in PBMCs with however a relatively wide range (mean 98, range 19-223 copies/10^5^ cells) [5]. In another series of 18 macaques (*Macaca tonkeana*), we previously found a very low viral load, ranging from 1 to 100 SFV DNA copies in 500 ng of peripheral blood buffy-coat DNA (equivalent to 0.7-70 SFV DNA copies /10^5^ cells) [18]. In chimpanzees, the situation in the buffy-coat was found to be quite similar [4].

In humans, Switzer et al. reported SFV infection (by an *Angolan colobus* or red-tailed monkeys strains) in 3 women from Democratic Republic of Congo, with viral loads ranging from 14-1755 copies/10^5^ blood cells [12]. In natural settings, our laboratory reported that viral loads range from 1 to 100 copies/10^5^ blood cells in 40 humans infected with a gorilla SFV strain, and 100-1000 copies/10^5^ blood cells in 2 individuals infected with a chimpanzee strain [10,11].

In this report, we extended these findings demonstrating low SFV DNA loads (less than 20 copies/10^5^ cells) in the same individuals, with variation possibly linked to sampling date and the use of PBMCs instead of buffy-coat.

Of note, putative fluctuation of SFV load should be considered with the potential for onward transmission if exposure occurs at that moment in time, even though epidemiological data, with no cases of human to human transmission reported so far, suggests this is not a major route of infection. We previously reported the case of BAD447, whose wife BAD460 was found to be positive for SFV by serology, but not by nested-PCR [11]. We note that SFV DNA loads are not especially high for this individual, either in PBMCs or in saliva. Further studies looking at SFV RNA and DNA loads in sexual compartments could be interesting.

#### Molecular characterization of SFV in the saliva of SFV-infected individuals

It is interesting that, if the blood is presumably the first site of SFV infection after a bite, 8/14 saliva samples also contained SFV DNA as shown by nested-PCR positivity. Detection and/or isolation of SFV in the buccal cavity have been shown in NHP and in 3 humans [6,14]. This could be due to the trafficking of PBMCs to the oral mucosa, contamination when sampling or infection of epithelial cells in the oral mucosa [19]. However, the genetic differences between SFV clones derived from samples of saliva and PBMCs as shown for LOBAK2 and BAD456 individuals suggest that SFV DNA found in the saliva does not simply mirror SFV DNA derived from PBMCs. Additional SFV cloning and sequencing experiments based on available material (BAD456 PBMCs and saliva samples) confirmed that the pools of clones of these two compartments were distinct, with a G/A polymorphism.

#### SFV intra-individual variability

The low intra-individual SFV variation is in agreement with the well-known genetic stability of SFV [20]. This is also in agreement with the low intra-individual SFV genetic diversity found in NHP [21]. Of note, inferred from intrinsic SFV RT fidelity [22], G/A polymorphism (accounting for 35% of *in vitro* RT substitution) in the G-G dinucleotide context (accounting for 27% of the A/T/G/C-G dinucleotide context) should represent 9% of the total viral genetic substitutions. The bias towards **G/A** polymorphism in a **G**G dinucleotide-context in SFV clones derived from BAD456 and LOBAK2 samples, which are absent in the available SFV gorilla strains from the same regions, could be indicative of APOBEC3 activity, as previously reported in few other individuals from another series [23]. It would have been of course interesting to have the sequence of the infecting gorilla SFV strain, which is obviously not possible to obtain, and to look at a more variable gene. This higher genetic variability allows to appreciate the compartmentalization of SFV in one individual. Indeed, SFV compartmentalization could also occur in other individuals but might not be visible, because the genetic variability of SFV clones is too low.

#### Detection of SFV DNA does not predict SFV replicative status

SFV DNA is not a dead-end product reminiscent of SFV primary infection and is still functional even decades after primary infection, as SFV has already been isolated from blood cells and saliva of SFV-infected humans [9], including from PBMCs of 2 hunters involved in this work [24]. This raises the question of the replication status of SFV within the human host. SFV DNA detected can come from proviral DNA (present in latent and productive infections) and/or viral DNA (present in productive infections). Indeed, SFV DNA can be integrated into the host genome (proviral DNA) and can also be produced upon productive infection (viral DNA), in a later reverse-transcription step. Production of viral DNA is preceded by a SFV RNA step during the life cycle of SFV [25]. In order to search for productive infection in humans, we investigated SFV RNA levels, in humans.

### Despite the presence of SFV DNA, is there any SFV active replication in infected humans?

In our work, no *pol* SFV RNA could be detected in human PBMCs and saliva, although we cannot exclude the expression of regulatory viral mRNA which is not detected by our assay.

SFV replicative cycles, considering either precoce or tardive reverse-transcription, require a SFV RNA step. SFV latency can be defined as presence of SFV DNA and absence of detectable SFV RNA. SFV latency in PBMCs was previously shown in some humans and NHP [6]. Furthermore, Boneva et al. found SFV DNA in samples of saliva derived from 3/7 SFV-infected individuals and could isolate the virus from buccal swabs in 1/6 cases [14]. In this case, virus isolation was successful from only one of four time points, suggesting that viral loads may fluctuate over time. This apparent higher restriction of SFV in human oral cavity is in contrast with what has been reported in macaques, where SFV replicates efficiently in the oral mucosa tissues (10^4^-10^9^ copies/10^4^ cells in the buccal swabs) [5]. Interestingly, SFV is readily transmitted via saliva among NHPs mainly after bites or grooming, whereas no secondary human transmission has been reported so far [8]. It is thus tempting to speculate that, in addition to obvious behavioral differences between humans and NHPs, absence or very low levels of SFV replication in human oral cavity tissues could prevent secondary transmission.

In macaques, transfusion experiments have shown a spread of SFV from blood to saliva with time [26]. Thus, absence of detectable SFV RNA in human saliva despite detection of SFV DNA could suggest a low-level infection of the buccal site.

The high replication rate of SFV *in vitro* (up to 10^7^ PFU/ml) [27] is in deep contrast with the apparent restriction of SFV in humans *in vivo*, which might suggest immune control of SFV. Infection of the oral cavity in humans might be restricted due to antiviral factors, non functional in the oral cavity of NHPs. Indeed, most restriction factors function in a species-specific manner [28]. Moreover, SFV induces type-I interferon (IFN-I) by human PBMCs [29] and is sensitive to several human IFN-I inducible factors [8].

To extend these results, it would be important to assess SFV levels in the other cohorts of SFV-infected persons. This could be done in individuals infected by SFV of macaques in Asia [30] and chimpanzees or *Cercopithecus* in Africa [11,31]. Furthermore, search for SFV RNA or SFV antigen expression in oral mucosa tissues of infected persons is necessary for further characterization of SFV infections in humans.

The investigation of viral cross-species transmission events, and their potential consequences for human health, include characterization of viral replication in humans compared to the reservoir host. Here, we demonstrate the presence of SFV DNA in samples of PBMCs and saliva derived from SFV-infected humans, but an absence of SFV RNA. It will be important to determine the cellular and molecular mechanisms involved in the control of SFV replication, especially in the oral mucosa tissues, as they might contribute to the absence of secondary transmission.

## Supporting Information

Figure S1 **Analysis of viral sequences from SFV-infected human BAD456.** Uncultured PBMCs and saliva samples from BAD456 hunter accidentally infected by a *Gorilla gorilla* SFV strain were analyzed for the presence of SFV sequences. A total of 18 and 20 different PCR-derived clones are depicted for PBMCs and saliva samples respectively. 20 PBMCs clones (blue) were grouped in "cluster 1" and 18 saliva clones (red) were grouped in "cluster 2" according to phylogenetic analysis (not shown). Their consensus sequences (PBMC-cons and SALIVA-cons, respectively) are also depicted. The 8 reference SFV sequences available in GenBank from *gorilla*s of the *Gorilla gorilla* species are aligned (green). 5 G-to-A mutations found in the hunter samples in a GG dinucleotide context and absent in the 8/8 *gorilla*s sequences are highlighted in blue. Sequences are aligned to the consensus sequence of PBMCs samples, which served as a reference due to its high similarity with gorilla strains. The 425bp-fragment of the pol-in sequence is shown. Due to the low SFV viral load, BAD456 clones of each cluster might derive from one to few *in* *vivo* SFV copies. Dots represent identical residues.
(PDF)

Figure S2 **Analysis of viral sequences from SFV-infected human LOBAK2.** Uncultured PBMCs and saliva samples from LOBAK2 hunter accidentally infected by a *Gorilla gorilla* SFV strain were analyzed for the presence of SFV sequences. A total of 18 and 20 different PCR-derived clones are depicted for saliva and PBMCs samples respectively. 11/20 PBMCs clones (dark blue) and 18/18 saliva clones (red) were grouped together in "cluster 1" and 9/20 PBMCs clones (light blue) were grouped in "cluster 2" according to phylogenetic analysis (not shown). Their consensus sequences (PBMC-cons1, SALIVA-cons and PBMC-cons2 respectively) are also depicted. The 8 reference SFV sequences available in GenBank from *gorilla*s of the *Gorilla gorilla* species are aligned (green). 4 G-to-A mutations found in a GG dinucleotide context and absent in the *gorilla*s sequences are highlighted in blue and 4 other mutations between PBMCs and saliva clones also present in some *gorilla*s sequences were highlighted in grey. Sequences are aligned to the consensus sequence of PBMC cluster1 samples, which served as a reference due to its high similarity with the *gorilla*s strains. The 425bp-fragment of the *pol-in* sequence is shown. Due to the low SFV viral load, LOBAK2 clones of each cluster might derive from one to few *in* *vivo* SFV copies. Dots represent identical residues.
(PDF)
