# Supplementary material for: Viral Latency in Blood and Saliva of Simian Foamy Virus-Infected Humans
Source: PLoS One. 2013 Oct 8;8(10):e77072. doi: 10.1371/journal.pone.0077072 (PMC3792900; doi:10.1371/journal.pone.0077072)
Supplement: Figure S1 — Analysis of viral sequences from SFV-infected human BAD456. Uncultured PBMCs and saliva samples from BAD456 hunter accidentally infected by a Gorilla gorilla SFV strain were analyzed for the presence of SFV sequences. A total of 18 and 20 different PCR-derived clones are depicted for PBMCs and saliva samples respectively. 20 PBMCs clones (blue) were grouped in "cluster 1" and 18 saliva clones (red) were grouped in "cluster 2" according to phylogenetic analysis (not shown). Their consensus sequences (PBMC-cons and SALIVA-cons, respectively) are also depicted. The 8 reference SFV sequences available in GenBank from gorillas of the Gorilla gorilla species are aligned (green). 5 G-to-A mutations found in the hunter samples in a GG dinucleotide context and absent in the 8/8 gorillas sequences are highlighted in blue. Sequences are aligned to the consensus sequence of PBMCs samples, which served as a reference due to its high similarity with gorilla strains. The 425bp-fragment of the pol-in sequence is shown. Due to the low SFV viral load, BAD456 clones of each cluster might derive from one to few in vivo SFV copies. Dots represent identical residues. (PDF) [file pone.0077072.s001.pdf]

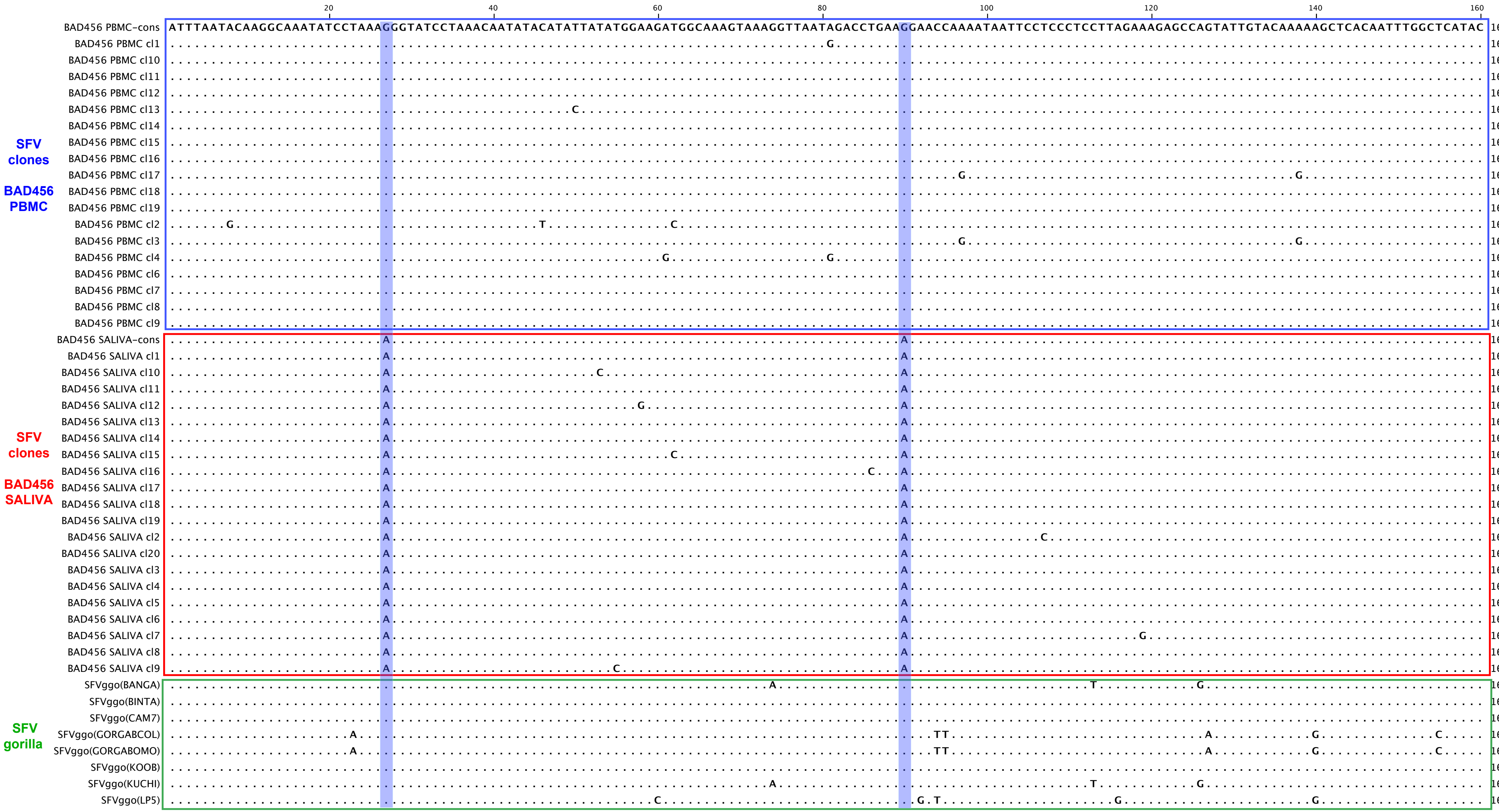

Figure S1: Analysis of viral sequences from SFV-infected human BAD456.

Uncultured PBMCs and saliva samples from BAD456 hunter accidentally infected by a *Gorilla gorilla* SFV strain were analyzed for the presence of SFV sequences. A total of 18 and 20 different PCR-derived clones are depicted for PBMCs (blue) and saliva (red) samples respectively. Their consensus sequences (PBMC-cons and SALIVA-cons, respectively) are also depicted. The 8 reference SFV sequences available in GenBank from gorillas of the *Gorilla gorilla* species are aligned (green). 5 G-to-A mutations found in the hunter samples in a GG dinucleotide context and absent in the 8/8 gorillas sequences are highlighted in blue. Sequences are aligned to the consensus sequence of PBMCs samples, which served as a reference due to its high similarity with gorilla strains. The 425bp-fragment of the *pol-in* sequence is shown. Due to the low SFV viral load, BAD456 clones of each cluster might derive from one to few *in vivo* SFV copies.

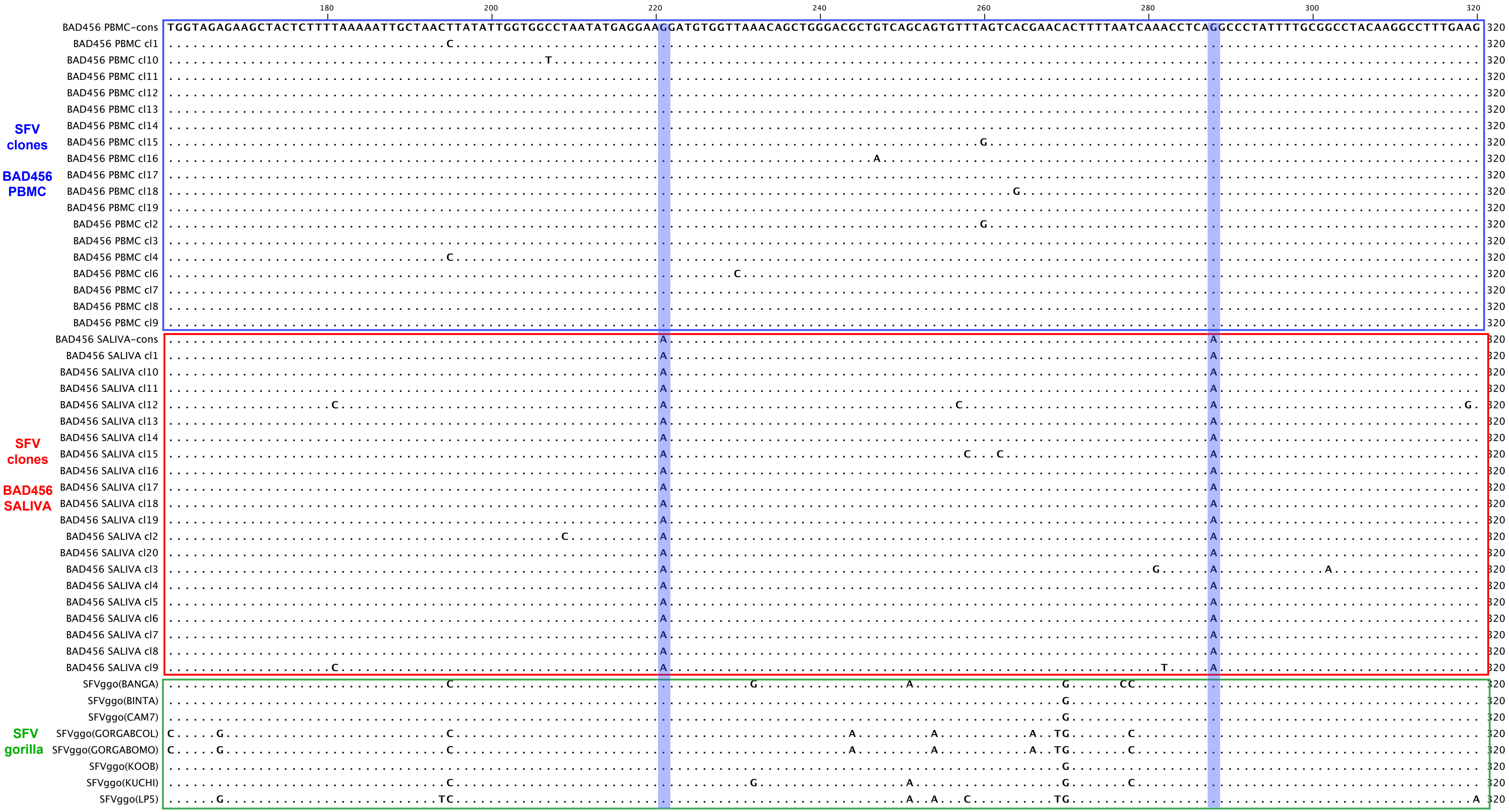
180
200
220
240
260
280
300
320
BAD456 PBMC-cons
BAD456 SALIVA-cons
SFV clones BAD456 PBMC
SFV clones BAD456 SALIVA
SFV gorilla
SFVggo(BANGA)
SFVggo(BINTA)
SFVggo(CAM7)
SFVggo(GORGABCOL)
SFVggo(GORGABOMO)
SFVggo(KOOB)
SFVggo(KUCHI)
SFVggo(LP5)

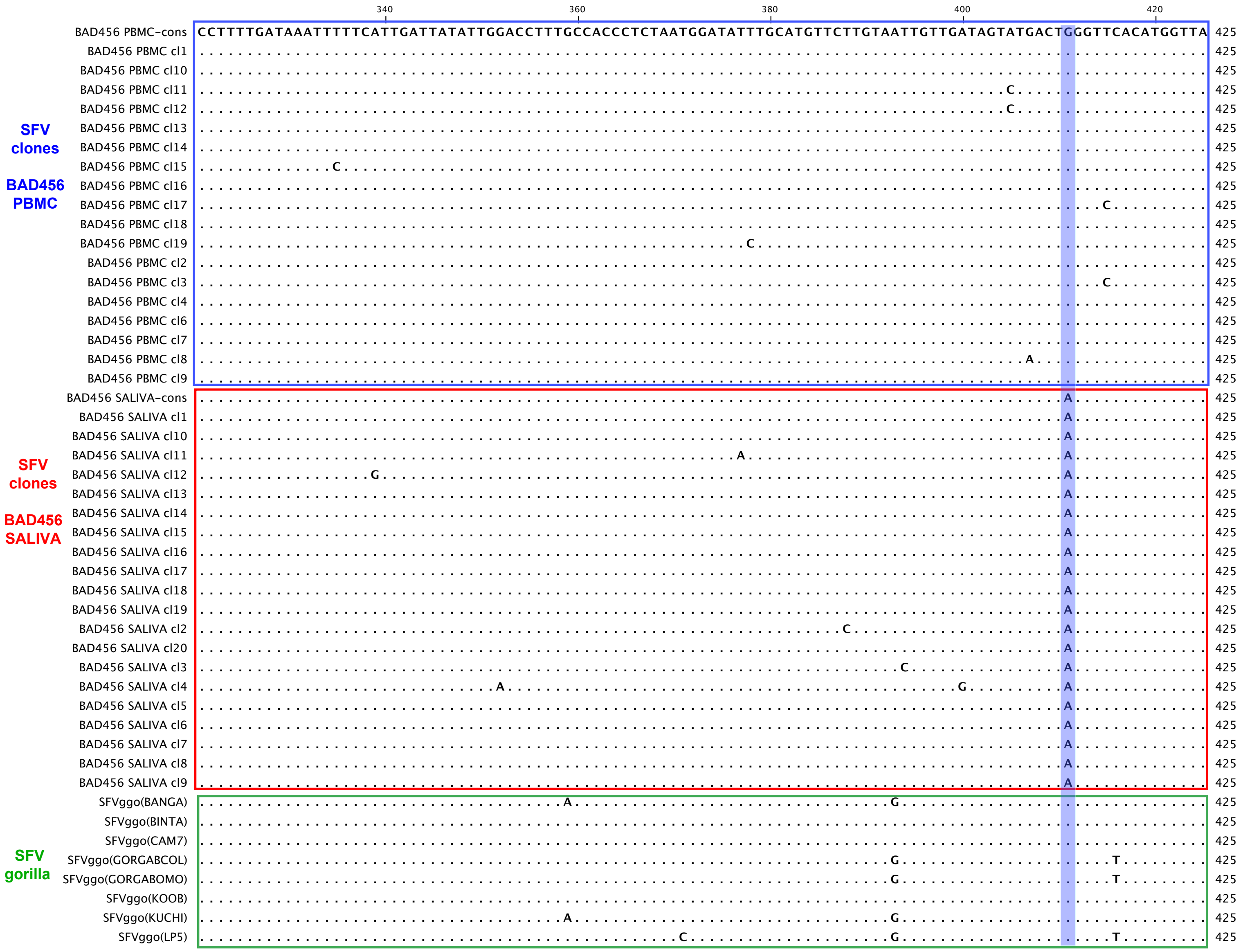
340
360
380
400
420
BAD456 PBMC-cons
CCTTTTGATAAATTTTTCATTGATTATATTGGACCTTTGCCACCCTCTAATGGATATTTGCATGTTCTTGTAATTGTTGATAGTATGACTGGGTTCACATGGTTA
425
SFV clones
BAD456 PBMC
SFV clones
BAD456 SALIVA
SFV gorilla
BAD456 SALIVA-cons
SFVggo(BANGA)
SFVggo(BINTA)
SFVggo(CAM7)
SFVggo(GORGABCOL)
SFVggo(GORGABOMO)
SFVggo(KOOB)
SFVggo(KUCHI)
SFVggo(LP5)
