# Supplementary material for: Viral Latency in Blood and Saliva of Simian Foamy Virus-Infected Humans
Source: PLoS One. 2013 Oct 8;8(10):e77072. doi: 10.1371/journal.pone.0077072 (PMC3792900; doi:10.1371/journal.pone.0077072)
Supplement: Figure S2 — Analysis of viral sequences from SFV-infected human LOBAK2. Uncultured PBMCs and saliva samples from LOBAK2 hunter accidentally infected by a Gorilla gorilla SFV strain were analyzed for the presence of SFV sequences. A total of 18 and 20 different PCR-derived clones are depicted for saliva and PBMCs samples respectively. 11/20 PBMCs clones (dark blue) and 18/18 saliva clones (red) were grouped together in "cluster 1" and 9/20 PBMCs clones (light blue) were grouped in "cluster 2" according to phylogenetic analysis (not shown). Their consensus sequences (PBMC-cons1, SALIVA-cons and PBMC-cons2 respectively) are also depicted. The 8 reference SFV sequences available in GenBank from gorillas of the Gorilla gorilla species are aligned (green). 4 G-to-A mutations found in a GG dinucleotide context and absent in the gorillas sequences are highlighted in blue and 4 other mutations between PBMCs and saliva clones also present in some gorillas sequences were highlighted in grey. Sequences are aligned to the consensus sequence of PBMC cluster1 samples, which served as a reference due to its high similarity with the gorillas strains. The 425bp-fragment of the pol-in sequence is shown. Due to the low SFV viral load, LOBAK2 clones of each cluster might derive from one to few in vivo SFV copies. Dots represent identical residues. (PDF) [file pone.0077072.s002.pdf]

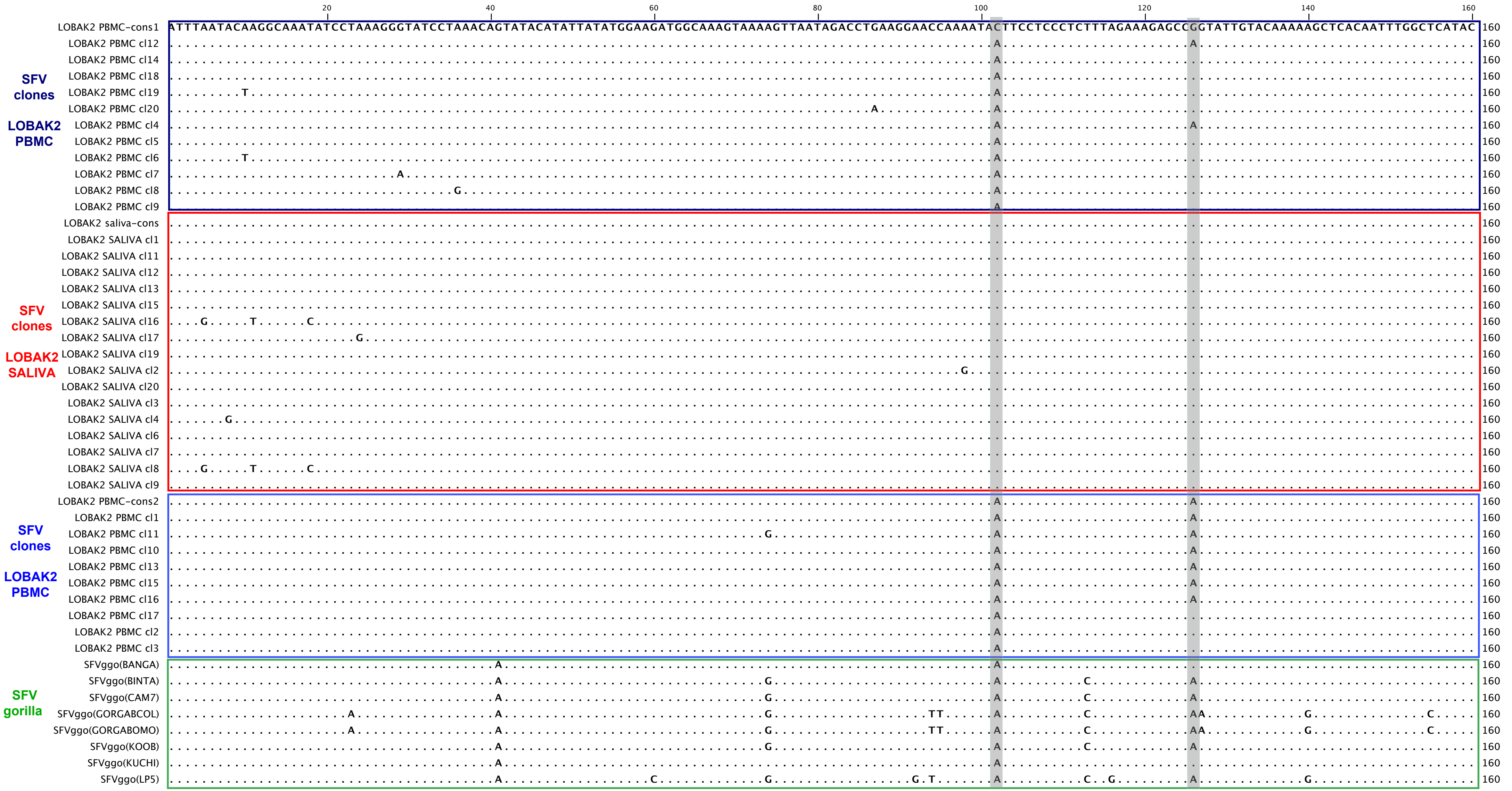

Figure S2: Analysis of viral sequences from SFV-infected human LOBAK2.
Uncultured PBMCs and saliva samples from LOBAK2 hunter accidentally infected by a *Gorilla gorilla* SFV strain were analyzed for the presence of SFV sequences. A total of 18 and 20 different PCR-derived clones are depicted for saliva (red) and PBMCs (blue) samples respectively. Their consensus sequences (PBMC-cons1, SALIVA-cons and PBMC-cons2 respectively) are also depicted. The 8 reference SFV sequences available in GenBank from gorillas of the *Gorilla gorilla* species are aligned (green). 4 G-to-A mutations found in a GG dinucleotide context and absent in the gorillas sequences are highlighted in blue and 4 other mutations between PBMCs and saliva clones also present in some gorillas sequences were highlighted in grey. Sequences are aligned to the consensus sequence of PBMC samples, which served as a reference due to its high similarity with the gorillas strains. The 425bp-fragment of the *pol-in* sequence is shown. Due to the low SFV viral load, LOBAK2 clones of each cluster might derive from one to few *in vivo* SFV copies.

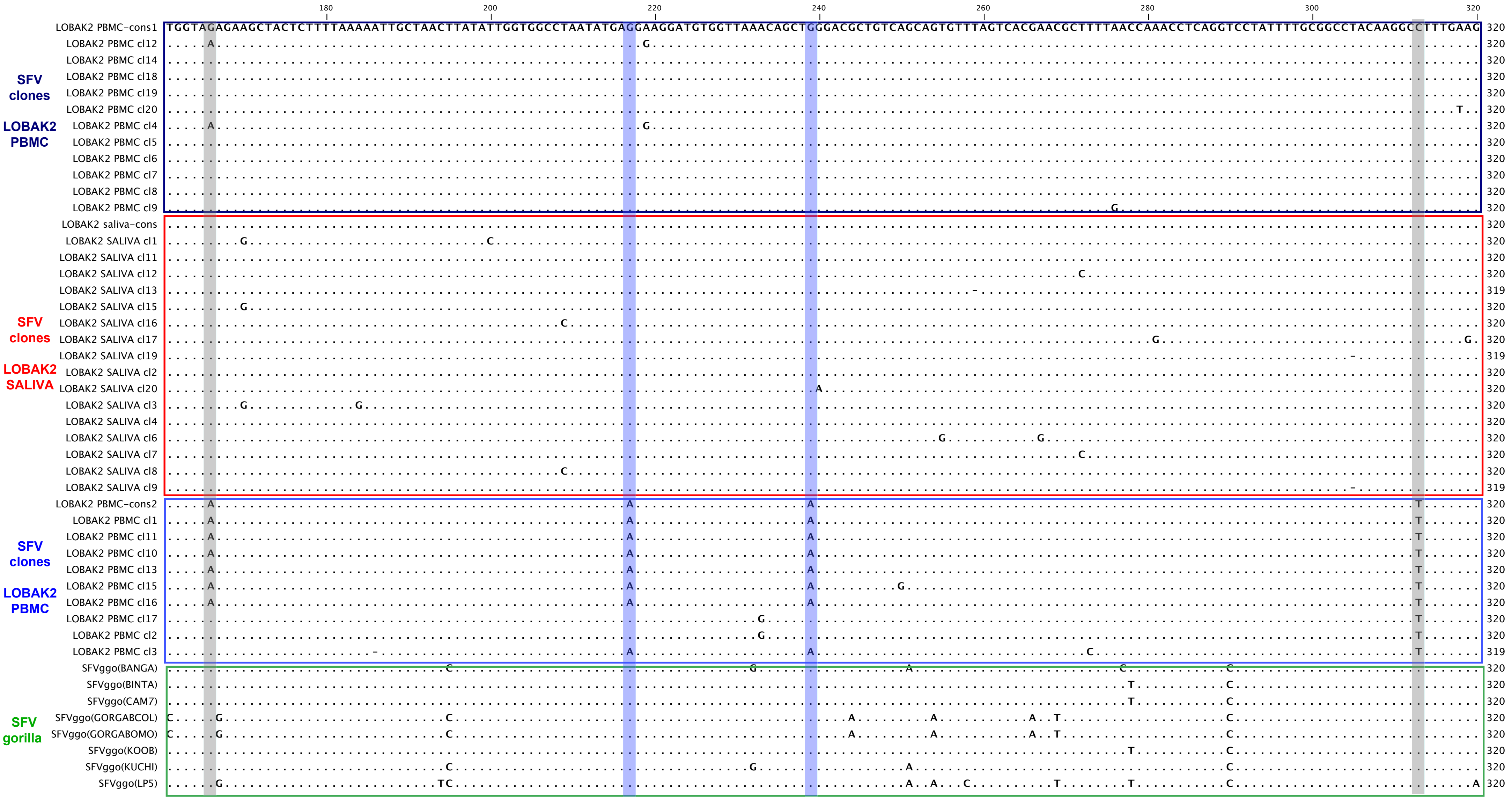

SFV clones LOBAK2 PBMC
SFV clones LOBAK2 SALIVA
SFV clones LOBAK2 PBMC
SFV gorilla
LOBAK2 PBMC-cons1
TGGTAGAGAAGCTACTCTTTTAAAAATTGCTAACTTATATTGGTGGCCTAATATGAGGAAGGATGTGGTTAAACAGCTGGGACGCTGTCAGCAGTGTTTAGTCACGAACGCTTTTAACCAAACCTCAGGTCCTATTTTGCGGCCTACAAGGCCTTTGAAG
LOBAK2 PBMC cl12
LOBAK2 PBMC cl14
LOBAK2 PBMC cl18
LOBAK2 PBMC cl19
LOBAK2 PBMC cl20
LOBAK2 PBMC cl4
LOBAK2 PBMC cl5
LOBAK2 PBMC cl6
LOBAK2 PBMC cl7
LOBAK2 PBMC cl8
LOBAK2 PBMC cl9
LOBAK2 saliva-cons
LOBAK2 SALIVA cl1
LOBAK2 SALIVA cl11
LOBAK2 SALIVA cl12
LOBAK2 SALIVA cl13
LOBAK2 SALIVA cl15
LOBAK2 SALIVA cl16
LOBAK2 SALIVA cl17
LOBAK2 SALIVA cl19
LOBAK2 SALIVA cl2
LOBAK2 SALIVA cl20
LOBAK2 SALIVA cl3
LOBAK2 SALIVA cl4
LOBAK2 SALIVA cl6
LOBAK2 SALIVA cl7
LOBAK2 SALIVA cl8
LOBAK2 SALIVA cl9
LOBAK2 PBMC-cons2
LOBAK2 PBMC cl1
LOBAK2 PBMC cl11
LOBAK2 PBMC cl10
LOBAK2 PBMC cl13
LOBAK2 PBMC cl15
LOBAK2 PBMC cl16
LOBAK2 PBMC cl17
LOBAK2 PBMC cl2
LOBAK2 PBMC cl3
SFVggo(BANGA)
SFVggo(BINTA)
SFVggo(CAM7)
SFVggo(GORGABCOL)
SFVggo(GORGABOMO)
SFVggo(KOOB)
SFVggo(KUCHI)
SFVggo(LP5)
180
200
220
240
260
280
300
320

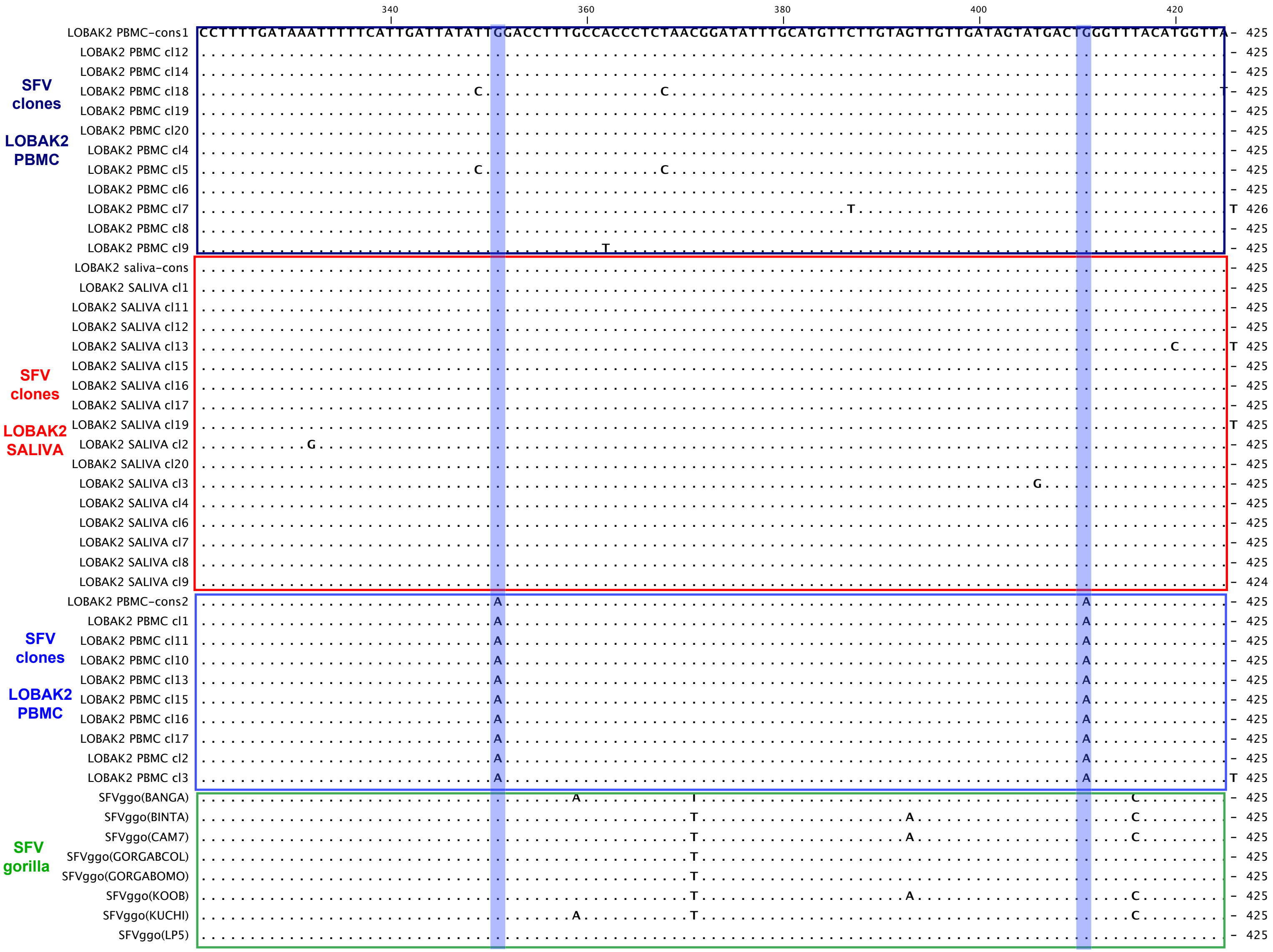
340
360
380
400
420
SFV clones LOBAK2 PBMC
LOBAK2 PBMC-cons1
LOBAK2 PBMC cl12
LOBAK2 PBMC cl14
LOBAK2 PBMC cl18
LOBAK2 PBMC cl19
LOBAK2 PBMC cl20
LOBAK2 PBMC cl4
LOBAK2 PBMC cl5
LOBAK2 PBMC cl6
LOBAK2 PBMC cl7
LOBAK2 PBMC cl8
LOBAK2 PBMC cl9
SFV clones LOBAK2 SALIVA
LOBAK2 saliva-cons
LOBAK2 SALIVA cl1
LOBAK2 SALIVA cl11
LOBAK2 SALIVA cl12
LOBAK2 SALIVA cl13
LOBAK2 SALIVA cl15
LOBAK2 SALIVA cl16
LOBAK2 SALIVA cl17
LOBAK2 SALIVA cl19
LOBAK2 SALIVA cl2
LOBAK2 SALIVA cl20
LOBAK2 SALIVA cl3
LOBAK2 SALIVA cl4
LOBAK2 SALIVA cl6
LOBAK2 SALIVA cl7
LOBAK2 SALIVA cl8
LOBAK2 SALIVA cl9
SFV clones LOBAK2 PBMC
LOBAK2 PBMC-cons2
LOBAK2 PBMC cl1
LOBAK2 PBMC cl11
LOBAK2 PBMC cl10
LOBAK2 PBMC cl13
LOBAK2 PBMC cl15
LOBAK2 PBMC cl16
LOBAK2 PBMC cl17
LOBAK2 PBMC cl2
LOBAK2 PBMC cl3
SFV gorilla
SFVggo(BANGA)
SFVggo(BINTA)
SFVggo(CAM7)
SFVggo(GORGABCOL)
SFVggo(GORGABOMO)
SFVggo(KOOB)
SFVggo(KUCHI)
SFVggo(LP5)
